# The Hospital World

**Published:** 1910-10-22

**Authors:** 


### THE HOSPITAL WORLD.

### Personalia.

As we are going to press we learn that Prince Francis of Teck is progressing favourably. It will be recalled that a few weeks ago the announcement was made that His Serene Highness had been advised to undergo an intranasal operation, which was successfully performed. This was for the relief of suppuration in the antrum, and the Prince's condition was much improved as a result of the operation. Later, however, he developed pleuro-pneumonia, and from this also he seemed to make a satisfactory recovery. A recurrence of pyrexia, however, was found to be associated with signs of pleural effusion; an exploratory puncture revealed the presence of an empyema, and this was then operated on by Mr. Pearce Gould. As chairman of the Weekly Board of the Middlesex Hospital His Serene Highness has, of course, only just completed the arduous and successful issue of the special appeal. No man in the hospital world will be more sympathised with in illness than the Prince.

---

Their Majesties the King and Queen have become patrons of the Royal Dental Hospital.

Mr. Alderman Ashworth has been elected chairman of the Bury Joint Hospital Board, in succession to Mr. Councillor Chambers, whose term is over.

Though Mr. Hazlerigg, late of the London Hospital, was appointed secretary to the West London Hospital, as announced in this column of our issue of August 6 last, he has not been able to take up his duties, having been offered a Government appointment in Ceylon. This offer was made to him before his appointment to the hospital had taken effect.

A vacancy on the board of management of the Norfolk and Norwich Hospital has been caused by the resignation of Mr. H. Newhouse. Mr. H. Newhouse, as was pointed out at the recent quarterly meeting of governors, has been a very good friend to the hospital and a very hard worker. The work he has done in itself constitutes a permanent record, and is typical of the immense use to an institution which a really capable member of its board can be. One of Mr. Newhouse's achievements has been the compilation of a map which shows the areas in which district presidents resided, and this, we are informed, has been most useful. Everyone regrets that Mr. Newhouse's connection with the board is now formally ended.

Everyone interested in the education of women, and more particularly in the education of women practitioners, will realise what a loss feminine medicine has suffered from the death of Mrs. Isabel Thorne. Having spent some early years of her life in the Far East, and having gained there an idea of the scope which awaited women doctors, she returned to England in 1868 and tried to become herself a qualified practitioner. It was a courageous attempt, for no examinations were open at that time to women medical students, in spite of the fact that Miss Garrett had succeeded in gaining the L.S.A. diploma. Mrs. Thorne, in company with other brave pioneers, Miss Chaplin Ayrton and Miss Edith Pechey among them, having been repulsed at Edinburgh, returned to the Metropolis with the bold determination to create for themselves what no one else would allow them to share—namely, a School of Medicine for Women. This attempt took more than thirty years to carry out, and would have taken longer had not Mrs. Thorne, backed by the active co-operation of Mr. Thorne, Mr. J. Stanfield, and her brother possessed no less tact than tenacity. She was hon. secretary to the school on its foundation, and added to her other laurels a genuine prize for pioneers in her election as the first of her sex to the Board of Management of the Royal Free Hospital. To-day the board has several women members, and they and their institutional fellow-workers throughout the country will long remember their first leader; for, however much the institutional scope for women may extend in the future, Mrs. Thorne was the true pioneer, and it is the first blow which demands the greatest determination and courage. We can only be glad that Mrs. Thorne lived to see the triumph of her efforts.

The Rev. Dr. Barrett has been elected to fill the vacancy which occurred on the board of management at Norwich. Other nominations were made, but Dr. Barrett gained the suffrages of the governors. As a governor himself, Dr. Barrett, in the words of Mr. Jewson, who supported his nomination, "has rendered the hospital such conspicuous service that we cannot do better than secure his more regular services week by week." Moreover, though this is his first election to the board, he has been asked to stand frequently, and has refused only because his clerical work has taken up too much of his energies to leave a margin for institutional work. Now, however, Dr. Barrett hopes to be more at leisure and to supplement his previous efforts on behalf of the hospital, as platform speaker, and as a secretary to the Hospital Saturday and Sunday Fund, by taking a share of the routine work which falls to the board of management. We feel sure that the Norfolk and Norwich Hospital will be grateful that Dr. Barrett's leisure has enabled him to come forward in this way.

---

### Elections and Appointments.

Mr. J. S. Battle has been elected to fill the vacancy on the weekly ward of the Lincoln County Hospital caused by the death of Mr. W. H. Morton.

The Rev. W. Adam and the Rev. R. W. Forbes have been elected directors of the Forfar Infirmary. Mr. Robertson has been reappointed president, and Mr. David Steele vice-president.

Mr. W. A. Sturdy, Mr. J. Bradford, J.P., and Captain Sergison, J.P., have been appointed additional trustees of the proposed King Edward and Eliot Memorial Hospital, Haywards Heath, for which the two latter gentlemen have presented a site.

Mr. J. Burder has been elected to fill the vacancy caused in the directorate of the Lewes Dispensary and Infirmary and Victoria Hospital, by the resignation of Lord Monk Bretton. Mr. F. Fawsett, the Rev. E. D. Stead, Mr. J. C. Robinson, and Mrs. Verrall have been re-elected.

